# Supplementary material for: Submicron-Sized Vermiculite Assisted Oregano Oil for Controlled Release and Long-Term Bacterial Inhibition
Source: Antibiotics (Basel). 2021 Oct 29;10(11):1324. doi: 10.3390/antibiotics10111324 (PMC8614931; doi:10.3390/antibiotics10111324)
Supplement: Supplementary file 1 [file antibiotics-10-01324-s001.zip › antibiotics-1401531-supplementary.pdf]

Supplementary Materials

Submicron-Sized Vermiculite Assisted Oregano Oil for Controlled Release and Long-term Bacterial Inhibition

Sukitha Geethma Kothalawala[1], Jun Zhang[1,*], Yue Wang[1], and Chengzhong Yu[1,2,*]

[1] Australian Institute of Bioengineering and Nanotechnology, University of Queensland, Brisbane City, QLD, 4072, Australia; s.kothalawala@uq.net.au (S.G.K.); j.zhang11@uq.edu.au (J.Z.); yue.wang1@uq.edu.au (Y.W.)

[2] School of Chemistry and Molecular Engineering, East China Normal University, 200241 Shanghai, China; czyu@chem.ecnu.edu.cn (C.Y.)

* Correspondence: j.zhang11@uq.edu.au, czyu@chem.ecnu.edu.cn

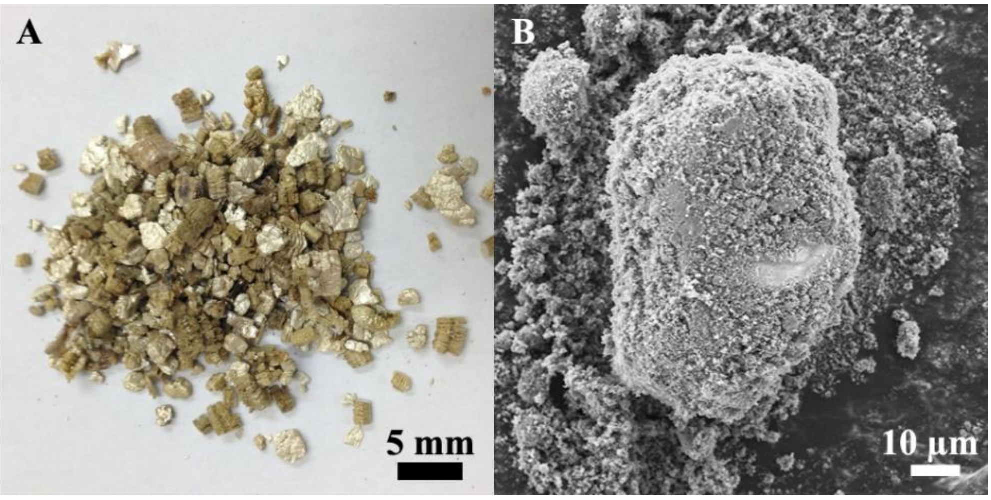

Figure S1. (**A**) Digital photo of RV, (**B**) SEM image of pre-milled vermiculite.

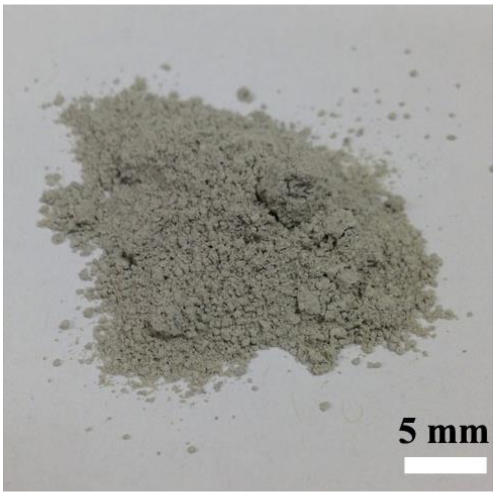

Figure S2. Digital photo of SMV.

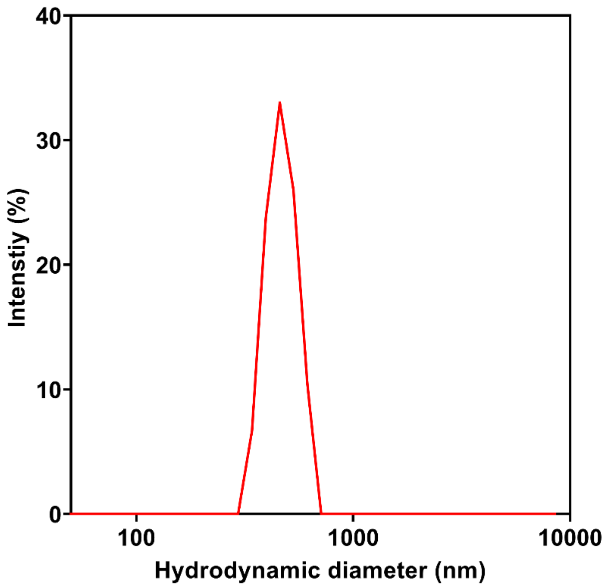

Figure S3. DLS size distribution of SMV in water.

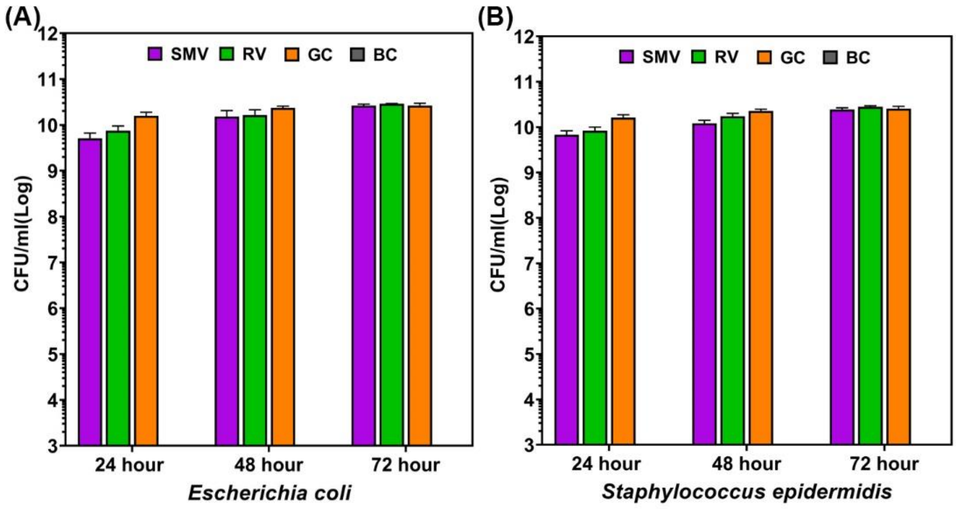

Figure S4. In vitro time-dependent bacterial inhibition test results of SMV and RV towards (A) *E. coli* (B) *S. epidermidis.*

Table S1. Elemental percentages from EDS analysis of SMV

Element	Atomic number	Mass percentage (%)	Atom percentage (%)	Absolute percentage error (%)
**K**	19	2.94	1.63	0.13
**Fe**	26	4.63	1.79	0.18
**Al**	13	2.01	1.61	0.10
**Si**	14	40.20	30.98	0.20
**Mg**	12	7.50	6.68	0.26
**Ti**	22	0.53	0.24	0.05
**O**	8	42.19	5.07	1.34
